# To evaluate the efficacy and safety of ^125^I seed implantation in SCLC as second line therapy

**DOI:** 10.1097/MD.0000000000029251

**Published:** 2022-07-22

**Authors:** Guoli Cheng, Yizong Wang, Dianguo Dong, Wenjie Zhang, Lei Wang, Zhaojun Wan

**Affiliations:** a Department of Oncology, Rizhao Hospital of TCM, Rizhao, Shandong, China; b Department of Respiration, Rizhao Hospital of TCM, Rizhao, Shandong, China; c Department of Surgery, Rizhao Hospital of TCM, Rizhao, Shandong, China; d Department of Oncology, People's Hospital of Rizhao, Rizhao, Shandong, China.

**Keywords:** ^125^I seed implantation, branchytherapy, overall survival, progression-free survival, small cell lung cancer

## Abstract

This study aimed to evaluate the efficacy and safety of iodine 125 (^125^I) radioactive seed implantation for small cell lung cancer at the limited stage of relapse as second line therapy. We collected 6 patients with recurred limited stage small cell lung cancer, who got pathological diagnosis after a bronchoscopic biopsy and then received standard first line treatment, combined chemotherapy and radiotherapy, including prophylactic cranial irradiation. These recurred small cell lung cancer patients got ^125^I seed implantation treatment as second line therapy, if the treatment not good responsive or the disease got rapid progress, we used the second line chemotherapy as salvage treatment. Clinical data of these patients were collected and short-term effects were observed. The follow-up period lasted for 42 months. All the patients tolerated the procedure of ^125^I radioactive seed implantation very well. We followed up the patients to 42 months. Five patients got complete remission and 1 patient got partial remission at 1 month after implantation. The objective response rate was 100%. The median survival time was 26 months. And median progression-free survival was 12 months after ^125^I treatment. And about the complications, 1 patient suffered from the light aerothorax, 1 patient had a little hemoptysis. Our study showed that ^125^I seed implantation as second line regimen in small cell lung cancer that recurred locally after first line treatment was effective and safe. That could improve the overall survival and progression-free survival only comparing to the second line chemotherapy. Therefore ^125^I seed implantation as brachytherapy protocol is a promising method and can be applied as second line treatment to control the locally recurred small cell lung cancer.

## 1. Introduction

Small cell lung cancer is a very highly malignant cancer, fast to grow, easy and early to disseminate, easy to relapse, sensitive to both chemotherapy and radiotherapy, but has bad prognosis. The 5 years survival rate is only <7%.^[1]^ Small cell lung cancer (SCLC) is usually classified into a 2-stage system, limited disease (LD), and extensive disease (ED) according to the Veterans Administration Lung Study Group (VALG) of America that was widely used now.^[2]^ Most patients are found in extensive stage and lose chance to get radical surgery. And even in limited stage many people could not accept operation or have a very high recurrence rate after radical surgery because of its potential transfer characteristics.^[3]^ So for patients with limited stage SCLC the standard treatment regimen is chemotherapy combining external radiotherapy.^[4]^ Prophylactic cranial irradiation can improve the 3-year survive. Despite high initial response rate, most patients eventually relapse. Except for topotecan, few treatment options then remain. Signaling pathways have been identified that might yield new drug targets.^[5]^ But, SCLC still has a bad prognosis. Hence, we still need to explore new methods. ^125^I radioactive seed implantation has been studied for many years and is widely used in many solid tumors like non-small cell lung cancer, prostate cancer, pancreatic cancer, liver cancer or brain cancer as supplementary therapy or palliative therapy,^[6]^ that has become a very important method. But for small cell lung cancer, not so much data to prove the efficacy and safety. Here we retrospectively analyzed 6 patients to prove that.

## 2. Patients and Methods

### 2.1. Patients

**Figure 1.**
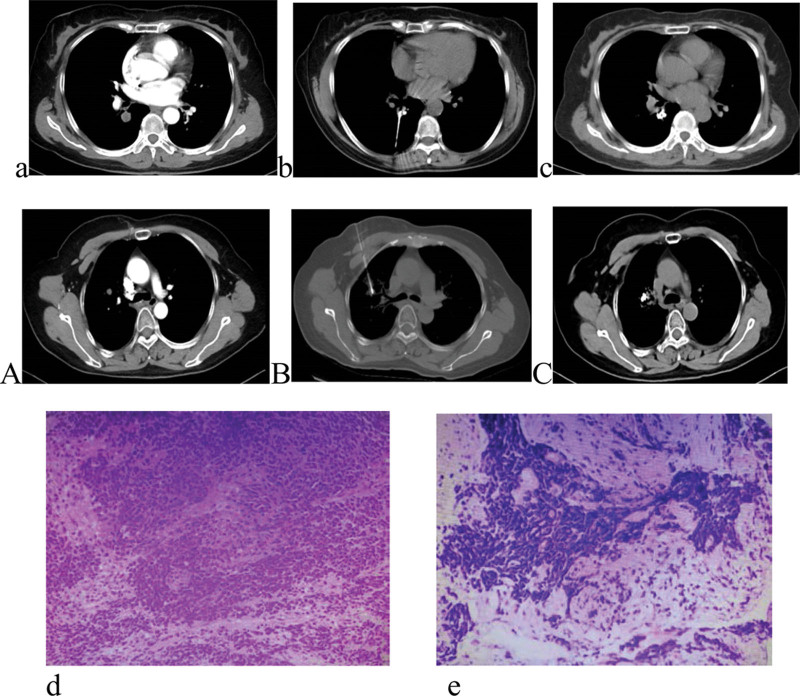
a. Representative image showing the tumor site and size in 1 patient. b. The procedure image of ^125^I seeds implantation in the patient. c. The image of the patient after 1 month. A. Representative image showing the tumor site and size in another patient. B. The procedure image of ^125^I seeds implantation in the patient. C. The image of the patient after 1 month. d. Representative pathological image of 1 patient. e. Representative pathological image of another patient.

A total of 6 patients who received ^125^I seed implantation from January 2014 to July 2019 in traditional Chinese medicine hospital of Rizhao city. This study was approved by the ethical committee of the hospital. All the patients were diagnosed with pathological results (Fig. 1) by bronchoscopic biopsy and with immunohistochemical support like Syn, CgA, and CD56 positive results. All these 6 patients, 52 to 67 years old (median age 59 years), 4 men, 2 women, were diagnosed in limited stage in initial diagnosis and got complete remission (CR) after the first line therapy, when they suffered from recurrence, the recurred lesion was only solitarily located in lung and the size of the solitary lesion was <3 cm. All the characteristics of these patients were listed in Table 1.

**Table 1:** Patient characteristics (n = 6).

Characteristic	Value
Median age (range)	59 (52–67)
Sex	4 males/2 females
Primary tumor stage (n)	limited stage(6)
Tumor size (preoperation, before implantation)	=3 cm
Other metastatic sites	No
ECOG	0–2
Previous regimen	EP (chemotherapy)+RT (radiotherapy)
Tumor marker (before implantation)	
Syn	5 cases high/1 case normal
Pro-GRP	5 cases high/1 case normal
Tumor marker (1 month after implantation)	
Syn	normal
Pro-GRP	normal
hyponatremia	none

Inclusion criteria were: patients with limited stage small cell lung cancer without chance to get resection and get complete remission after chemotherapy and radiotherapy as first line treatment. And they suffered from recurrence, the recurred lesion was only solitarily located in lung and the size of the solitary lesion was <3 cm. And the ECOG score was <2. The case records were complete. Patients who could not tolerate puncture because of severe basic disease were excluded. According to the inclusion criteria we collected 6 patients for the study. And every patient was informed consent. Some patients were excluded because case records were incomplete or following up was not timely or standard. Those included 3 cases.

### 2.2. Treatment process and complications

Contrast computed tomography (CT) scan before ^125^I seed implantation was prepared to recognize the relationship between tumor and vessels, according to the CT image, the total volume of each tumor was calculated using a treatment planning system (TPS). Radioactive particles were purchased from Tianjin Said Biological Pharmaceutical Co, LTD (Tianjin, China). Iodine-125 seed source was a sealed source for radionuclide. The parameters of ^125^I seed were as follows: half-life 59.6 days; tissue penetration 1.7 cm; half value layer 0.025 mm lead; activity 0.6 × 0.8 mCi. Selected particle activity (individual particle surface activity of 0.6 mCi) and a prescription dose (120 Gy) were fed into the TPS system, the implant channel was designed, and the number of particles was calculated. Before treatment the patients got blood examination including CBC, blood coagulation, and electrolytes etc. Besides, we gave the patient codeine phosphate tablet in case of severe cough that would result in increasing the incidence rate of aerothorax and other complications.

When everything was prepared, the therapeutic intervention was performed in the CT room. All patients underwent local disinfection anesthesia before ^125^I seed implantation. And then used 18G needle to puncture at the predetermined point and angle according to the preoperative plan under CT-guidance. When the needle reached the edge of the tumor lesion and determined the position and angle of needle by CT scan. Using a seed implant machine to perform seed implantation retrogradely and keep the interval distance 0.5 to 1.5 cm. During the procedure, neural structures and large vascular structures were carefully avoided. Giving CT scan again after operation and compared with the TPS planning to check the prescription dose and the real distribute dose. If necessary, complement the needed seeds again. When completing the procedure, performed CT scan again to make sure whether there were aerothorax, hemothorax, seed displacement, subcutaneous emphysema, and so on. From the CT scan, only 1 patient had a little aerothorax. Puncture site was bandaged and compressed to achieve hemostasis after the procedure. On the whole, every patient tolerated the procedure very well and went back to the ward safely. All patients remained under observation at the hospital for 1 or 2 full days.

### 2.3. Follow-up and clinical effect evaluation

One month after the implantation, patients were revisited to determine whether re-implantation was required based on the evaluation and tumor size alteration as well as whether complications were present like seed displacement or radioactive radiation pneumonitis, etc. And then all the patients were revisited at 3 months, 6 months after implantation. Hereafter, we followed up the patients every half a year until the end time of the study (September 2021). The items were followed up including the physical examination, CT Scan, and blood panel when patients came back for review every time. We recorded the data of every patient and calculated the PFS1 (from the first CR to the first progression), PFS2 (from iodine 125 seed implantation to the second progression), objective response rate, overall survival (OS), and OS2 (from iodine 125 seed implantation to death or the last visiting time or to the end time of study [for still alive]). The follow-up continued to 3.5 years.

Tumor treatment response was determined following the World Health Organization criteria 6: CR was defined as the complete disappearance of the lesion, which lasted for >4 weeks. Partial remission (PR) was defined as a reduction in the tumor size for >50% and remained unchanged for 4 weeks. Stable disease (SD) was defined as a reduction in tumor size <50% or an increase in the size <25%. Progressive disease (PD) was defined as an increase in the tumor size of at least 25%. The sum of CR+PR was used to calculate the overall response rate.

### 2.4. Statistical analysis

Progression-free survival (PFS) and OS were the primary endpoints of this study. Data were calculated using the Kaplan–Meier method in SPSS 13.0 software (SPSS).

## 3. Results

### 3.1. Tumor control

**Figure 2.**
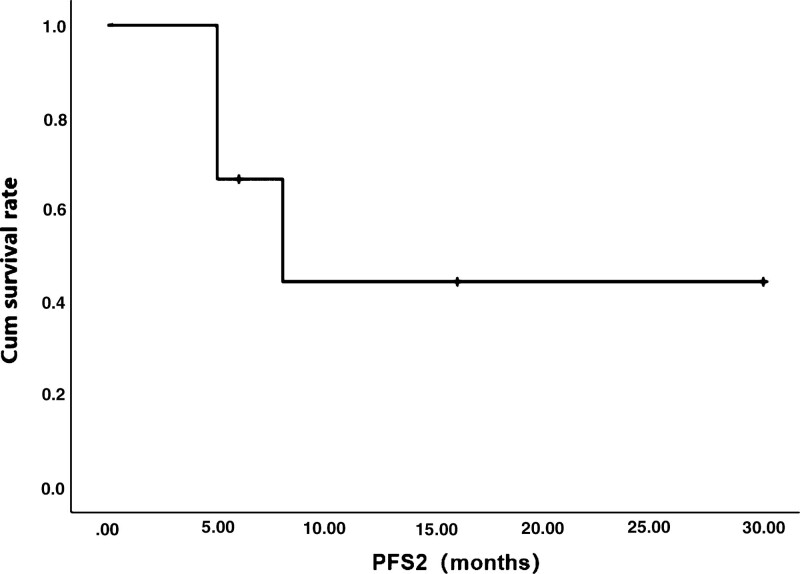
Kaplan–Meier curve of progression-free survival 2 (PFS2) of patient, months after ^125^I seed implantation.

**Figure 3.**
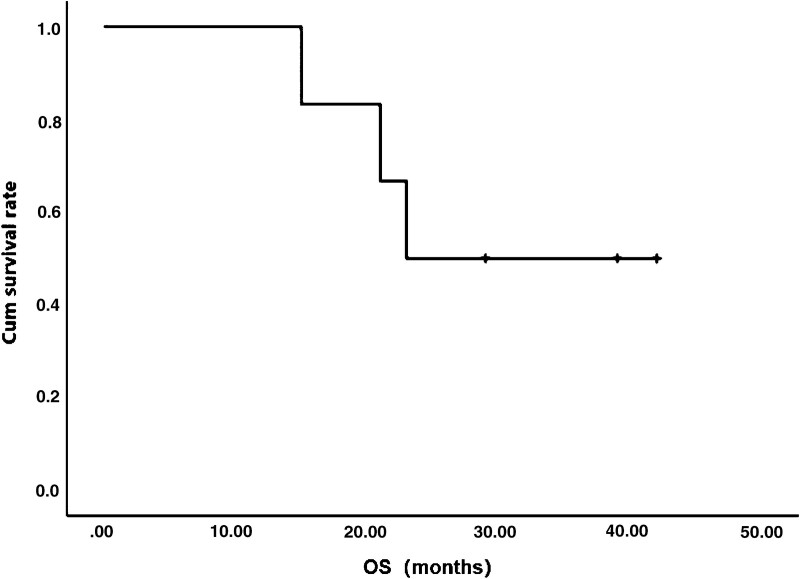
Kaplan–Meier statistical analysis of overall survival (OS).

After 1 month of ^125^I seed implantation, the outcomes of CT examination showed that 5 patients had complete disappearance of the tumor lesion (CR), 1 patient had PR. The percentage of tumor control in 6 patients (CR or PR) was 100% (Table 2). All of the 6 patients were followed up to 42 months, and none of them was lost during the follow-up. The median overall survival time (mOS) of the 6 patients was 26 months and median overall survival time (mOS2) was 12 months. The median progression-free survival time (mPFS) was 7 months. Up to September 2021, 2 out of the 6 patients had survived for the whole 42-month follow-up. The survive curves by Kaplan–Meier method were showed below (Figs. 2 and 3).

**Table 2:** Recent evaluation of efficacy after operation.

	N = 6				
Time	CR	PR	SD	PD	ORR (CR+PR, %)
1 month after operation	5	1	0	0	100
3 months after operation	4	1		1	83.3
6 months after operation	3			3	50

CR = complete remission, ORR = objective response rate, PD = progressive disease, PR = partial remission, SD = stable disease.

### 3.2. Complications

One patient had a little pneumothorax and 1 patient had sputum blood, they both recovered without treatment. None of the patients had any severe complications such as bleeding or heavy aerothorax. Long-term complications such as particle-migration or radioactive radiation pneumonitis were not presented. And also no other complicates were found during the follow-up period.

## 4. Discussion

Small cell lung cancer is a highly aggressive malignant tumor of neuroendocrine origin, strongly associated with smoking tobacco and accounting for 15% of all lung cancers.^[7]^ Small-cell lung cancer is considered limited-stage if it is still within the chest or extensive-stage if it has spread outside the chest.^[8]^ Currently, chemotherapy and radiation therapy are recommended for treatment of limited-stage small-cell lung cancer if it is localized and has not spread outside of one side of the chest. Current treatment guidelines recommend platinum-based chemotherapy plus thoracic radiotherapy for the treatment of limited stage SCLC, and chemotherapy alone for extensive disease, with prophylactic cranial irradiation.^[9]^ These recommendations are based on the premise that SCLC disseminates early and is very chemo-sensitive. The high throughput sequencing has deepened our understanding of its biology. The arrival of immune checkpoint inhibitors revolutionizes the management of extensive disease. Atezolizumab in combination with chemotherapy as a first-line treatment also demonstrated improved efficacy in the IMpower133 study.^[10]^ This is the first phase III study to achieve an improvement OS in >30 years for extensive stage SCLC. But the treatment of localized disease has changed little.^[11]^

For limited stage SCLC, combined chemotherapy and concurrent or sequential radiotherapy are the standard treatment as we said before. Even the patients have chance to get radical resection, they still need to accept chemotherapy and radiotherapy. Therefore, when the limited stage SCLC patients suffer from recurrence after first line therapy that treatment got complete remission, they also need to accept chemotherapy that will be chosen according to the interval time from the first line regimen. But for the patients with localized recurrence, maybe we can use local methods to control the disease to prolong the survival time and avoid the patient from adverse effect that will be brought by the second chemotherapy. Of course, if the patients get extensive metastasis we plan to give them chemotherapy as rescue regimen. As we know, iodine 125 seed implantation has been used in many solid tumors widely in the world in recent years and become more mature.^[12]^ Iodine 125 particle implantation therapy is an effective method and is available to achieve truly synchronous chemo-radiotherapy, which belongs to internal radiotherapy, its local dose is much higher than external radiation, and therefore iodine 125 can improve the local control rate. To interfere with DNA synthesis of tumor cells by emitting continuous low energy gamma rays, iodine seed produces killing effect at the late mitotic stage.^[13]^ Its effective irradiation distance is only 1.7 cm, so it can produce high dose in the local tumor and low dose in the normal tissue.

So in our study, we tried to retrospectively analyzed 6 patients to determine the efficacy and safety of ^125^I seed implantation. From our results we can see that: after 1 month of ^125^I seed implantation, 5 cases had complete remission and 1 case had PR. The percentage of tumor control was 100% (Fig. 1). All of the 6 patients were followed up to 42 months, the median overall survival time (mOS) of the 6 patients was 26 months and media overall survival time (mOS2) was 12 months. The media progression-free survival time (mPFS) was 7 months. Up to September 2021, 2 out of the 6 patients had survived for the whole 42-month and still had very good quality of life. Besides, to the last followed time, all the patients did not present severe complications. Compared with the date of 4 to 5 months median survive time,^[8]^ iodine 125 seed implantation in our study can improve the survive time very well. So for the patients with locally recurred SCLC, especially for those who refuse to receive chemotherapy or could not tolerate chemotherapy because of severe complications like neutropenia or leukopenia, iodine 125 seed implantation can be a choice. But, the patients we choose were only locally recurred cases and the size of lesion was smaller than 3 cm, that was optimal choice. So the results just can represent a part of patients with SCLC and further study with more cases should be explored again.

## 5. Conclusions

In summary, our study showed that ^125^I seed implantation as second line therapy in small cell lung cancer that recurred locally after first line treatment was effective and high safe. That could improve the overall survival and progression-free survival. Therefore ^125^I seed implantation as brachytherapy protocol is a promising method and can be applied as second line treatment to control the locally recurred small cell lung cancer. That may be a suitable and optional method.

## Author contributions

Conceptualization: Haibin Wang.

Data curation: Zhaojun Wan.

Formal analysis: Lei Wang.

Investigation: Dianguo Dong.

Investigation: Lei Wang.

Resources: Yizong Wang.

Software: Zhaojun Wan.

Visualization: Wenjie Zhang.

Writing – original draft: Guoli Cheng.
